# Psychosis in a Patient with Muscular Dystrophy : Case Report and Literature Review

**DOI:** 10.1192/j.eurpsy.2023.1628

**Published:** 2023-07-19

**Authors:** O. Ali, H. Raai

**Affiliations:** Psychiatry, SBH Health System, New York, United States

## Abstract

**Introduction:**

Knowledge about muscular dystrophies and in particular X-linked inherited disorders such as Duchenne and Becker Muscular Dystrophy has been gradually acquired as more research studies have been conducted to better understand the pathogenesis, management and prognosis of these conditions. However, little is known about a probable correlation between muscular dystrophies and neuropsychiatric disorders. We present the case of a 41-year-old male with history of a mild rare form of muscular dystrophy and elevated CPK level, who presents with psychotic symptoms that resolve with proper management of the underlying medical condition.

**Objectives:**

The purpose of this case report is to emphasize the importance of being mindful when diagnosing patients with a psychiatric illness as psychotic symptoms could also result from underlying rare muscular dystrophies.

**Methods:**

A comprehensive review of literature using databases, such as PubMed and Google Scholar was conducted to gain a better understanding of this specific disorder and to rule out conditions that present in a similar way. Keywords used were Muscular Dystrophy, Elevated CPK, Psychosis, Becker, Duchenne.

**Results:**

Data shows that patients with these muscular dystrophies have mutations that affect Dp71 expression. Moreover, Dp71 is expressed in postsynaptic membranes in the glutaminergic pathway whose alteration might contribute to neuropsychiatric disorders. As there is growing body of evidence of rhabdomyolysis encountered in patients treated with antipsychotics, there is less data available about a possible causal relationship between rhabdomyolysis and subsequently elevated CPK inducing psychosis.

**Conclusions:**

Further studies could be helpful to explore a possible correlation between an elevated CPK level and psychotic symptoms as demonstrated by this case report. A thorough history taking, psychiatric and medical evaluations would prevent misdiagnosis of psychiatric disorders and would lead to proper management of these rare cases.

**Disclosure of Interest:**

None Declared

